# Society Notes

**Published:** 1897-02

**Authors:** H. Scheppergrell

**Affiliations:** Chairman Southern Section; New Orleans


### Society Notes.

### The American Laryngological, Rhinological and Otological Society.

NEW ORLEANS, Oct. 20, 1896.

DEAR DOCTOR:—The meeting of the Southern Section of the American Laryngological, Rhinological and Otological Society will be held in New Orleans, March 3d and 4th, 1897. This date has been selected, as it will permit visiting members to see New Orleans during the carnival season, and will enable them to secure half-rate railroad transportation.

We shall be pleased to have you attend the meeting and to receive from you the title of a paper, on a subject within the object of the Society, to be read before the meeting of the Southern Section.

The number of physicians, who devote their attention to diseases of the ear, nose and throat, has increased so much, and the subjects for discussion have become so extensive, that it is difficult, in the time devoted to the annual meeting of the Society, to give the necessary time to many important questions. On this account, the four sections of the Society have been formed, the object of which is to promote the interest of the speciality during the interval of the annual meets.

A meeting of a Laryngological, Rhinological and Otological Society in the South, is a distinctly new enterprise, and should be encouraged. With the active co-operation of the physicians interested in this work, the Carnival meeting of the Southern Section of the American Laryngological, Rhinological and Otological Society will be an assured success.

Candidates for membership should send their names, properly endorsed, to Dr. Robt. C. Myles, Secretary, 46 West 38th St., New York, or to Dr. W. Scheppegrell, so that they may be acted upon by the Council of the Society.

Very truly yours, H. SCHEPPERGRELL,
Chairman Southern Section.

